# Local mechanical stimuli correlate with tissue growth in axolotl salamander joint morphogenesis

**DOI:** 10.1098/rspb.2022.0621

**Published:** 2022-05-25

**Authors:** Ester Comellas, Johanna E. Farkas, Giona Kleinberg, Katlyn Lloyd, Thomas Mueller, Timothy J. Duerr, Jose J. Muñoz, James R. Monaghan, Sandra J. Shefelbine

**Affiliations:** ^1^ Serra Húnter Fellow, Department of Physics, Universitat Politècnica de Catalunya (UPC), Barcelona, Spain; ^2^ Department of Mathematics, Laboratori de Càlcul Numeric (LaCàN), Universitat Politècnica de Catalunya (UPC), Barcelona, Spain; ^3^ Department of Mechanical and Industrial Engineering, Northeastern University, Boston, MA USA; ^4^ Department of Biology, Northeastern University, Boston, MA USA; ^5^ Department of Bioengineering, Northeastern University, Boston, MA USA; ^6^ Institute for Chemical Imaging of Living Systems, Northeastern University, Boston, MA USA; ^7^ Centre Internacional de Mètodes Numèrics en Enginyeria (CIMNE), Barcelona, Spain; ^8^ Institut de Matemàtiques de la UPC-BarcelonaTech (IMTech), Barcelona, Spain

**Keywords:** synovial joint development, transient receptor potential vanilloid 4, cartilage mechanosensitivity, poroelasticity, cartilage growth

## Abstract

Movement-induced forces are critical to correct joint formation, but it is unclear how cells sense and respond to these mechanical cues. To study the role of mechanical stimuli in the shaping of the joint, we combined experiments on regenerating axolotl (*Ambystoma mexicanum*) forelimbs with a poroelastic model of bone rudiment growth. Animals either regrew forelimbs normally (control) or were injected with a transient receptor potential vanilloid 4 (TRPV4) agonist during joint morphogenesis. We quantified growth and shape in regrown humeri from whole-mount light sheet fluorescence images of the regenerated limbs. Results revealed significant differences in morphology and cell proliferation between groups, indicating that TRPV4 desensitization has an effect on joint shape. To link TRPV4 desensitization with impaired mechanosensitivity, we developed a finite element model of a regenerating humerus. Local tissue growth was the sum of a biological contribution proportional to chondrocyte density, which was constant, and a mechanical contribution proportional to fluid pressure. Computational predictions of growth agreed with experimental outcomes of joint shape, suggesting that interstitial pressure driven from cyclic mechanical stimuli promotes local tissue growth. Predictive computational models informed by experimental findings allow us to explore potential physical mechanisms involved in tissue growth to advance our understanding of the mechanobiology of joint morphogenesis.

## 1. Background

The shape of a synovial joint is critical to its functionality in movement and locomotion. Joint morphogenesis in the developing vertebrate limb bud follows a well-known sequence of events [1]. First, the mesenchymal cells forming the early limb bud differentiate into chondrocytes, except for those in the interzone, where the future joint will appear. Through a process known as cavitation, the skeletal rudiments are physically separated and the synovial cavity is formed. During this sequence of events, chondrocyte proliferation and matrix production in the rudiment result in tissue growth and final joint shape. Movement-induced mechanical stimuli condition the correct formation of joints throughout this morphogenetic stage [2,3]. Yet, how motion and biophysical forces influence joint shape is not fully understood to date [4,5].

Animal studies using immobilized chicks [6–10], reduced-muscle and absent-muscle mice [11–13] and paralysed zebrafish larvae [14] have shown that reduced and restricted muscle contractions during embryonic development results in skeletal abnormalities, including alterations in joint shape. Elucidating the role of motion in joint development is challenging in animal models that develop *in ovo* or *in utero* [3]. An animal model that allows rigorous control of the biophysical environment during joint morphogenesis will further our understanding of how mechanical stimuli are linked to the shaping of the joint. Axolotl salamanders (*Ambystoma mexicanum*) regenerate limbs throughout life by recapitulating developmental processes. Regenerating axolotl limbs undergo stereotypical patterns of gene expression and cell differentiation that resemble mammalian joint development [15,16]. Their limbs are morphologically similar to human limbs, with elbow joints comparable in cellular composition and skeletal structure to mammalian synovial joints [17,18]. Despite these similarities, observations in axolotls are not directly transferable to mammals given the differences in level of activity, mechanical stimulation and overall timing of the process in regenerating axolotl limbs. Nonetheless, insights into how chondrocyte response to mechanical stimuli during axolotl joint morphogenesis regulates joint shape may have application in the broader study of how movement affects skeletogenesis [5]. Limb regeneration has been extensively characterized at the tissue and cellular level, but to our knowledge, no studies have investigated the role of muscle-induced loading in salamander joint regeneration to date.

Joint morphogenesis is driven by local growth of the cartilage tissue that forms the bone rudiments. Different theories have been proposed to explain how growth occurs, including proliferation and subsequent hypertrophy, migration and intercalation of cells [19]. To bring about such behaviours, chondrocytes respond to mechanical stimuli like changes in osmotic pressure, cellular stretch, or fluid shear [20]. Ion channels, integrin signalling and the primary cilia are all known mechanosensors that initiate intracellular signalling cascades ultimately resulting in the transcription, translation, and/or molecular synthesis that leads to cartilage tissue growth [20–22]. *In vitro* studies have shown that the transient receptor potential vanilloid 4 (TRPV4) channel is possibly a key transducer of biophysical stimuli to regulate cartilage extracellular matrix (ECM) production [23–25]. TRPV4 activation in chondrocytes has been linked to osmolarity changes in *in vitro* studies [26,27]. Recent studies have shown it also responds to physiologic levels of strain loading [28,29], although there is also evidence to the contrary [30,31].

To identify the specific mechanical stimuli influencing joint shape, computational models can help decipher the role of biophysical stimuli in tissue growth and joint morphogenesis. Techniques like finite element analysis (FEA) are specially suited to studying the mechanics of morphogenesis. They allow for the quantitative, unbiased testing of the biophysical mechanisms that might be regulating and controlling morphogenesis [32,33]. A few studies have used FEA to examine how changes in mechanical loading affect joint morphogenesis [34–37]. These models demonstrated shape changes based on generic joint shapes and idealized loading conditions in two dimensions. The computational models assume that dynamic hydrostatic compression promotes cartilage growth, which is in line with experimental studies that have shown an increase in ECM production with cyclic compression [38–42]. Yet, these numerical studies use a static approximation via linear elasticity. As such, they are unable to intrinsically capture the effects of dynamic loading on cartilage, including the fluid flow and extracellular pressure to which cells probably respond. To better comprehend how local mechanical stimuli drive the shaping of the joint, we must model the tissue as a poroelastic medium, which incorporates a fluid component to account for the dynamic changes in pressure and velocity of extracellular fluid present in cartilage.

The goal of this study was to determine the role of chondrocyte mechanosensitivity on joint morphology, and identify potential mechanisms by which mechanical loading is translated into unequal tissue growth that results in joint shape. Experiments on regenerating axolotl limbs provided information on how impairing the TRPV4 channel affects chondrocyte proliferation and the shaping of the joint. Through the computational modelling of cartilage growth, we sought to link TRPV4 desensitization to an altered transduction of physical stimuli. Predictive computational models informed by the experimental findings allowed us to explore potential physical mechanisms influencing joint morphogenesis.

## 2. Effect of transient receptor potential vanilloid 4 desensitization on regrowing axolotl elbow joints

Most known genetic disfunctions of the TRPV4 channel resulting in skeletal dysplasias are related to a gain of function [43,44]. The lack of regulation of intracellular calcium ions induced by the chemical activation of TRPV4 channels means that the chondrocytes lose their mechanosensitivity and are effectively unable to detect and respond to mechanical stimuli [45,46]. Hence, with the aim of restricting the ability of cells to respond to mechanical stimuli during joint morphogenesis, we used the TRPV4 agonist GSK1016790A in regrowing axolotl forelimbs. To identify the effect of TRPV4 desensitization on the shaping of the joint, we quantified bone rudiment morphology of the regrown limbs. Chondrocyte proliferation was also measured to study its role in local tissue growth during joint morphogenesis.

### (a) Axolotl experiments

Larval animals (3–5 cm) were bilaterally amputated just proximal to the elbow joint. GSK1016790A was reconstituted in dimethyl sulfoxide (DMSO) and injected intraperitoneally at 50 μg kg^−1^ at 22 days post amputation (dpa, *n* = 6). Control animals (*n* = 6) were injected with 50 μg kg^−1^ DMSO. Injections were repeated at 48 h intervals. At 32 dpa, all animals were injected intraperitoneally with 5-Ethynyl-2’-deoxyuridine (EdU) and L-Azidohomoalanine (AHA). Limbs were collected 18 h later, fixed and stained.

**Figure 1.**
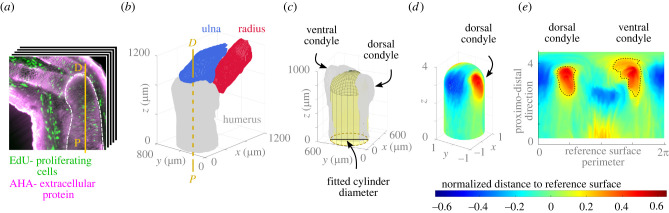
Overview of the experimental data analysis pipeline applied to a representative fully-regrown control limb. (*a*) Three-dimensional light sheet images of the axolotl elbow were aligned to the proximo-distal (P-D) axis of the humerus and (*b*) oriented in three-dimensional space. (*c*) A reference surface (yellow) was used to (*d*) map the perpendicular distance from the humerus surface to the reference surface and normalized to the cylinder diameter. (*e*) The mapped values were flattened out. A threshold value of 0.2 was considered to define the contour of the condyles (dashed line). (Online version in colour.)

We imaged nascent macromolecule synthesis in the regenerated forelimbs with light sheet fluorescence microscopy following the whole-mount click-it-based technique in Duerr *et al.* [47]. EdU is incorporated into newly synthesized DNA, which allowed for the quantification of cell proliferation through EdU-positive nuclei segmentation. AHA enabled the visualization of chondrocyte protein translation, most likely ECM, which provided a well-defined outline of the bone rudiment’s perichondria. Quantification of the three-dimensional shape was then possible through the analysis of the humerus outline (figure 1*a*).

Electronic supplementary material, figure S1A illustrates the timeline of the experiments and electronic supplementary material, figure S1B shows an example of the animal size used, and the location of the amputation. Injections started at 22 dpa, which is roughly when joint cavitation occurs in regenerating limbs in 3–5 cm sized animals, and continued throughout the joint morphogenesis stage of the joint formation process until 30 dpa. It is difficult to ascertain complete penetration of GSK101 via intraperitoneal delivery. Treated animals showed a systemic response to injections as they appeared debilitated and lethargic, though there was no visible difference in size of the animals or limbs.

Electronic supplementary material, figure S1C shows a central slice of a three-dimensional image stack obtained for an exemplary control elbow at 33 dpa. No ossification was observed in the fully regrown limbs; all bone rudiments were cartilaginous at this final stage. All light sheet images were acquired using a Zeiss light sheet Z.1 microscope paired with Zen software.

### (b) Experimental data analysis

We segmented the regrown bone rudiments (33 dpa), identified the proximo-distal longitudinal axis of the humerus and ulna through computation of the minimum principal axis using the Fiji plugin BoneJ [48], and then aligned all limbs in three-dimensional space (figure 1*b*). The alignment process included mirroring of right limbs so that all limbs had the dorsal and ventral condyles in the same relative position in space. A cylinder was fitted to the aligned humerus surface using the Matlab [49] File Exchange function ‘cylinderfit’ (a regression modelling tool), and a hemispherical cap was placed on top to create the reference surface (figure 1*c*). These were shifted vertically upwards until the hemispherical cap was tangent to the distal end of the humerus surface. The distance from the reference surface to the humerus surface was mapped onto the reference surface and normalized using the fitted cylinder diameter (figure 1*d*) to account for animals of different size. We quantified dorsal and ventral condyle shapes and sizes based on the corresponding normalized areas and normalized volumes, respectively, which were extracted from the two-dimensional standardized representation of the humerus surface (figure 1*e*).

To quantify proliferating cells, we manually generated a small training set to train the deep learning algorithm Stardist3D [50], which was used to identify the EdU-stained cell nuclei in the three-dimensional image stack. The Fiji plugin three-dimensional Objects Counter [51] was used on the cell nuclei masks produced by Stardist3D to identify proliferating cell positions and volumes. Outliers were removed based on cell volume and we used a fixed-length cut-off to ensure quantification of cell proliferation was performed in an equivalent humerus volume across different limbs.

Electronic supplementary material, figure S2 provides a visual summary of the complete workflow, which was implemented using a combination of Fiji [52], the ZeroCostDL4Mic implementation of Stardist3D [53] and a customized code in Matlab [49]. We grouped all limb results for each measurement and ran a Shapiro–Wilk normality test. Except for the proliferating cell count, all other data measurements were normally distributed. A one-way ANOVA was used to check for statistically significant differences between the control (*n* = 10) and GSK101 (*n* = 11) groups of normally distributed data. The proliferating cell count *p*-value was obtained using a Kruskal–Wallis test.

### (c) Experimental results

**Figure 2.**
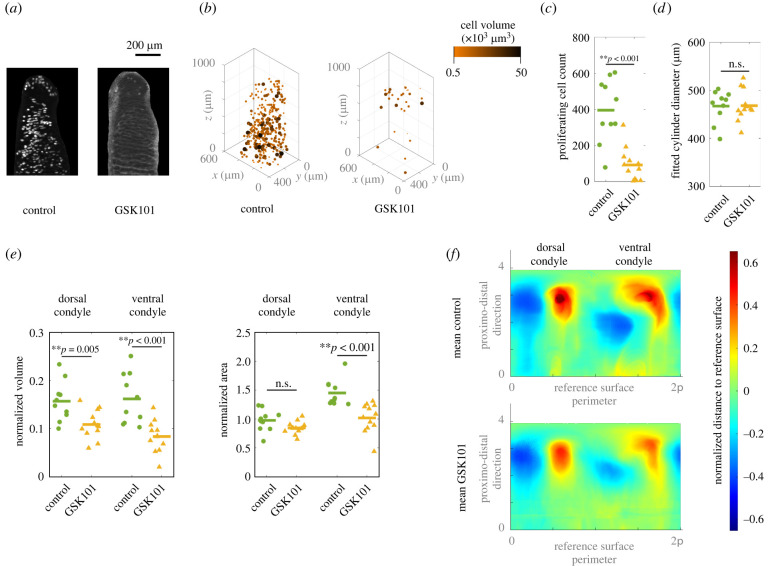
Quantification of humerus growth and shape in regenerated axolotl humeri. (*a*) Light sheet image of an EdU-stained humerus for a representative humerus from each group and (*b*) corresponding three-dimensional cell nuclei positions. (*c*–*e*) Results of the statistical analysis on the data points. The volume and area of the condyles (*e*) are normalized with the diameter of the cylinder fitted to each humerus shaft (*d*). (*f*) Mean two-dimensional surface maps for each group. Measurements were obtained following the methodology outlined in figure 1. (Online version in colour.)

Our results reveal significant differences in cell proliferation and bone rudiment shape between the humeri of the control group and the GSK101 group. A central slice of a representative EdU-stained humerus from the control and GSK101 groups (figure 2*a*) and the three-dimensional distribution of the cell nuclei identified for each (figure 2*b*) depict the differences in cell proliferation between groups. A central slice of each humerus analysed is provided in the electronic supplementary material, figure S3. The mean value of the proliferating cell count in the humeri of the control group is fourfold that of the GSK101 group (*p*-value < 0.001; figure 2*c*). However, the diameter of the humeri shaft is similar for both groups (figure 2*d*). To ensure this is not owing to an insufficiently sensitive measurement method, we computed additional metrics using an alternative methodology (electronic supplementary material, S4). All measures of humeri shaft size computed indicate there is no significant differences between the two groups.

The normalized volumes of both dorsal and ventral condyles are larger for the control group than the GSK101 group (*p*-value = 0.005 and <0.001, respectively). The normalized areas of the ventral condyles in the control group are also larger (*p*-value < 0.001), while no significant difference was found for the normalized areas of the dorsal condyles (figure 2*e*). These findings are visually reflected in the mean humerus surfaces computed (figure 2*f*), where a darker shade of red in the condyles of the mean control humerus indicates more prominent condyles for this group with respect to the GSK101 group. To compute the mean two-dimensional surface maps, we aligned the individual two-dimensional surface maps (electronic supplementary material, figure S5) based on the position of the dorsal condyle centroids.

## 3. Computational predictions of joint morphogenesis

To explore whether the changes in humerus morphology owing to TRPV4 desensitization observed in experiments can be attributed to impaired tissue mechanosensitivity, we created a finite element model of a regenerating humerus. We used this model to explore potential movement-induced mechanical stimuli as drivers of tissue growth during joint morphogenesis. The humerus bone rudiments in experiments had fully cartilaginous epiphyses. Cartilage tissue has a water content of roughly 80% by volume of tissue mass [54]. The mechanism for transduction of mechanical forces in tissues is not completely understood, but fluid flow is known to play an important role [20]. Poroelastic theory is commonly used in finite-element models of cartilage response to loading [55–58] because it can explicitly capture the fluid flow effects.

### (a) Modelling cartilage tissue growth within a poroelastic framework

The biphasic approach defines tissue as a mixture of an elastic solid skeleton with free-flowing fluid circulating within its pores. In cartilage, the fluid can be assimilated to the interstitial fluid in the tissue, i.e. water and dissolved ions, growth factors and other molecular components. The solid component represents the proteoglycans and collagen of the ECM and chondrocytes. Chondrocyte proliferation, hypertrophy, migration and/or intercalation as well as ECM production in cartilage can then be modelled together at tissue level through continuum growth of this solid phase (electronic supplementary material, figure S6). Following a common approach in the field [33–35,59], we consider growth rate to be a sum of biological and mechanical contributions.

The biological contribution represents the intrinsic morphogenetic biological factors that globally mediate tissue growth. Similar to past studies of joint morphogenesis [34,35], we assumed it is proportional to chondrocyte density in the bone rudiments. However, unlike these studies, our experimental measurements of chondrocyte density in a regenerating axolotl humerus revealed an approximately constant value throughout the bone rudiment at this stage of regrowth (electronic supplementary material, figure S7). Chondrocyte density quantification of the humeri in our experiments was not possible based on the AHA staining (electronic supplementary material, figure S9). We assumed a constant biological growth rate in time and space, within the humerus geometry and throughout the whole simulation time period in all our simulations. This implies cell density is the same in both groups.

The mechanical contribution is a function of the selected mechanical stimulus locally driving tissue growth. Mechanical loading is known to modulate the synthesis of ECM in chondrocytes. Collagen and aggrecan production, the main components of ECM in cartilage, depends on the magnitude, duration and type of loading. In particular, *in vitro* experiments have shown that cyclic compression promotes ECM production while static loading either has no effect on collagen and aggrecan levels, or inhibits cartilage growth [38–42]. Our poroelastic model is able to capture the differences between static and dynamic loading by defining mechanical growth proportional to a dynamic variable linked to the movement-induced fluid flow. We selected pore pressure of the fluid component, a hydrostatic measure akin to the hydrostatic stress used in past models, as the mechanical stimulus.

The discretized governing equations and continuum growth model were implemented in the open source finite element library deal.II [60]. The code used in this study is an extension of the poro-viscoelastic numerical framework in [61]. Growth was implemented following the algorithm in the electronic supplementary material, figure S8. Further details of the poroelastic formulation, the growth model and their numerical implementation are provided in the electronic supplementary material, S6.

### (b) A finite element model of joint morphogenesis

We generated a finite element model of a generic humerus bone rudiment after cavitation, i.e. at the start of the experiments, with the goal of predicting the grown humerus shape at the end of the joint morphogenesis stage. Given that our model is a tool to probe potential mechanisms of load mechanotransduction in joint morphogenesis, we strove to keep its parameters as generic as possible.

**Figure 3.**
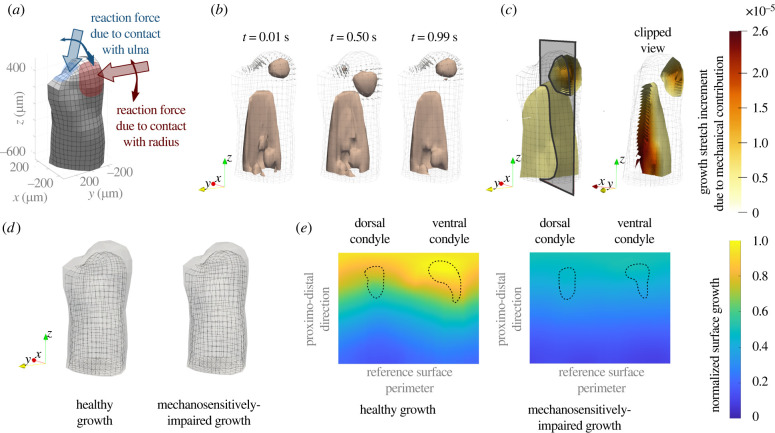
Computational predictions of joint morphogenesis considering pressure-driven local tissue growth. (*a*) Finite element model of the humerus simulating a flexion-extension cycle of the elbow. (*b*) Predicted pressure contour of 1 kPa at the start, middle and end of a flexion-extension cycle. (*c*) Local tissue growth owing to the mechanical contribution at the end of one cycle. (*d*) Grown humerus shape representing a healthy case (left) and a mechanosensitively impaired case (right), which used constant biological growth only. (*e*) Quantification of grown humerus shapes based on the normalized surface growth. (Online version in colour.)

The geometry and loading conditions (figure 3*a*) were informed by experimental data. A normally regenerating forelimb at 17 dpa in a 3 cm sized animal, which corresponds to the time point just after joint cavitation, was used (electronic supplementary material, figure S10A). We segmented the bone rudiment shapes from the three-dimensional image stack (electronic supplementary material, figure S10B).

A mesh was generated based on the smoothed-out surface of the segmented humerus with a total of 512 hexahedral elements. We scaled the geometry size to achieve a cross-sectional humerus size closer to the values identified in our experiments. Meshing of the geometry inevitably entails a slight loss of surface detail. We computed and visually compared the two-dimensional surface maps of both the segmented geometry and the meshed geometry (electronic supplementary material, figure S10C) following a procedure analogous to the one used in the humerus three-dimensional shape analysis. Comparison of two-dimensional surface maps confirmed that the meshed surface retained the main characteristics of the original humerus.

Free-flow boundary conditions across all external surfaces except the bottom (proximal) one were set in the finite element model. Vertical displacements of the bottom surface were fixed, and lateral displacements of nodes in the bottom surface were fixed (electronic supplementary material, figure S10D). These boundary conditions allowed for outward growth of the humerus shaft while avoiding spurious translations as well as the rotation of the whole bone rudiment.

The loading conditions applied (figure 3*a*; electronic supplementary material, figure S10E), modelled a 1 s flexion-extension cycle of the elbow. The growth resulting from a single cycle was extrapolated for multiple cycles. Loading was applied as a pressure over a roughly circular surface representing the contact areas between the radius/ulna and the humerus. A sine-like loading profile over this area was considered, with the loading area sweeping over the humerus surface. The sweep path was estimated based on anatomical observations of the axolotl elbow joint. The value of the load profile changed throughout the cycle to mimic the effect of muscle contractions, reaching the maximum value for the peak flexion position. Load step increments of 0.01 s were applied. We studied the effect of varying loading and boundary conditions on our computational results (electronic supplementary material, figure S11). In this way, we ensured the robustness of our computational set-up to produce results from which to extract meaningful insights.

The material properties were either estimated from the literature or based on an educated guess, except for the initial intrinsic permeability of the biphasic material. Preliminary simulations identified this parameter as having a considerable impact on the predicted patterns, while the rest of the material properties did not substantially alter the predicted growth patterns. Hence, we adjusted the value of the initial intrinsic permeability based on experimental stress-relaxation data obtained through nanoindentation tests on an axolotl forelimb (electronic supplementary material, figure S12). Electronic supplementary material, S9 provides further details of all model parameters.

### (c) Computational results

A regenerating humerus model based on local changes in fluid pressure (figure 3*b*) induced by an elbow flexion-extension loading cycle predicted a final humerus morphology (figure 3*d*, left) that resembled our experimental observations of the control group. When the mechanically driven growth component (figure 3*c*) was removed, shape prediction (figure 3*d*, right) resulted from constant volumetric biological growth only and was in accordance with the experimental observations of the GSK101 group. When the mechanically driven growth component was included, local mechanical growth occurred in regions of high compressive pressure, which were observed underneath the surface load representing the radius contact area throughout the cycle. However, we did not observe an analogous pressure below the load representing the ulna contact area. Pressure was most pronounced at the posterior proximal part of the humerus shaft. Complete predicted patterns for pressure as well as other mechanical stimuli that were initially considered as potential drivers of the mechanical growth model are provided in the electronic supplementary material, S10.

To quantify the differences between the healthy and mechanosensitively impaired cases, we computed at each surface node the magnitude of the distance between the original surface and the grown surface, and normalized this measure with the maximum value of the two cases. We then mapped the resulting patterns onto a reference surface, fitted to the original surface mesh, and flattened it to obtain a two-dimensional representation of normalized growth (figure 3*e*). The mapping procedure was analogous to the one used to obtain the two-dimensional surface maps of the experimental humeri (figure 1*c*–*e*). In both predictions, humerus surface growth increased towards the distal portion of the bone rudiment, but the healthy growth case resulted in larger values as well as a notably asymmetrical pattern. A larger surface growth was predicted in the area corresponding to the future ventral condyle for the healthy growth case (figure 3*e*, left). The contour of the condyles from the corresponding mean experimental surfaces in figure 2*f* is shown on the two-dimensional maps.

## 4. Discussion

### (a) Transient receptor potential vanilloid 4 desensitization during joint morphogenesis altered final humerus shape

Our analysis of the regenerating axolotl limbs revealed an altered humerus morphology for the GSK101 group (figure 2*c*–*e*). The mean two-dimensional surface maps computed for each group (figure 2*f*) illustrate the main findings: the condyles of the control animals have larger normalized volumes than the GSK101 group (darker shade of red in contour map). The shape of the ventral condyle, as measured based on the normalized area, is more affected by TRPV4 desensitization than the dorsal condyle.

We also analysed the shapes and sizes of the anterior and posterior concavities (blue regions in the two-dimensional surface maps; electronic supplementary material, figure S5) following an analogous procedure to the condyle measurements and did not observe significant differences between groups for any measurement. It could signify that these shape characteristics of the humerus were already present at the onset of the experiment; starting treatment with GSK101 sooner after amputation may result in more severe changes. We analysed a regenerating limb after cavitation (start of our experiments) for the purposes of developing the initial computational model. The two-dimensional surface map obtained (electronic supplementary material, figure S10C, top) supports the notion that the basic humerus shape could be present already at this stage. The concavities seem to already be present and the dorsal condyle is clearly defined, similar in shape to those of the fully regenerated limbs in the experiments (electronic supplementary material, figure S5). However, the ventral condyle is barely discernible after cavitation. This implies that the concavities and dorsal condyle may form in the earlier stages of the joint formation process, which is probably why we found little change in their shapes.

Taken together, this data indicates that TRPV4 desensitization during joint morphogenesis in regrowing forelimbs alters the final humerus shape. During regeneration, blastema cells dedifferentiate and may assume a different role in the regrowing limb. These cells have been shown to retain distinct roles in axolotls [62], which could affect the way chondrocytes in regenerating limbs respond to mechanical stimuli. However, joint morphogenesis occurs at a much later time point than the dedifferentation process within the limb regrowth timeline. It seems reasonable to assume that mechanotransduction pathways in joint formation of regenerating axolotl limbs are probably the same as those in developing limbs.

Numerous studies have shown that chondrocytes have several separate but overlapping mechanotransduction pathways [28,30]. Other channels of the TRP family have been suggested to have load-associated effects in cartilage [63], but TRPV4 is undoubtedly the major regulator of mechanical and osmotic signal transduction in this family. The Piezo1 and Piezo2 channels have also been identified as key stretch-induced mechanotransducers in chondrocytes [64]. It would be interesting to see whether altering these other channels has effects on morphology similar to those seen in this study, to further explore the interrelated roles of each channel in cartilage mechanotransduction. Furthermore, axolotls have much larger cell sizes and longer cell cycles than the vast majority of vertebrates, which probably influences their mechanosensitive response, and would also be a fascinating topic for further study.

Alternative ways of blocking mechanics in developing joints have been used in the past to study the effect of mechanical stimuli on joint morphogenesis, namely muscle paralysis in chicks [6–10] and genetically modified altered-muscle mice [11–13]. These studies also revealed morphological differences. Here, we used a TRPV4 agonist, which represents the clinical genetic deficits associated with abnormal skeletal development [65,66]. Our three-dimensional analysis of the humerus surface allows the assessment of shape changes that are not evident in more simple measures used in the past, such as cross-sectional outlines or linear anatomic measurements like humeral head width.

### (b) More prominent condyles and increased chondrocyte proliferation were not associated with larger humeri

The substantial reduction in cell proliferation of the GSK101 group (figure 2*a*–*c*; electronic supplementary material, figure S3) did not result in smaller humeri sizes (figure 2*d*). Axolotls have long cell cycles, which have been recorded to be up to 88 hours in regenerating tissues [67,68]. Throughout the 10-day experimental treatment, few complete cycles would have occurred. Also, proliferating cells were only a relatively small percentage of the total chondrocytes in the bone rudiment. Therefore, the total amount of cell proliferation may not have been sufficient to produce actual changes in bone rudiment size. In addition, our quantification of cell proliferation corresponded to an 18 h window at the end of the experiment, which may not be representative of the complete treatment period of 10 days.

The decrease in condyle normalized volumes and in the ventral condyle normalized area for the GSK101 group may be owing to matrix production instead of cell proliferation. Proliferation was not localized to the condyles, rather it was homogeneously distributed. Our data seem to indicate that TRPV4-mediated proliferation is unlikely to be a major contributor to growth during axolotl joint morphogenesis in regenerating forelimbs.

### (c) Local fluid pressure may promote tissue growth during joint morphogenesis

To link the experimentally observed changes in humerus morphology owing to TRPV4 desensitization with impaired mechanosensitivity in the growing tissue, we built a computational model of joint morphogenesis. Through hypotheses and simplifying assumptions, we have isolated a potential contributor to the mechanotransduction of mechanical loading into local tissue growth and subsequent shaping of the joint.

The computational results show that compressive fluid pressure can predict humerus morphology during joint morphogenesis. In the predicted normalized surface growth map for the healthy growth case (figure 3*e*, left) the ventral condyle exhibited a considerably larger amount of growth than the dorsal condyle, while the mechanosensitively impaired case (figure 3*e*, right) showed similar (smaller) growth values for both condyles. This agrees with the larger normalized area observed in the ventral condyle of the experimental control group with respect to the GSK101 group (figure 2*f*). The predictions for the healthy growth case (figure 3*e*, left) exhibited more growth towards the distal area than the mechanosensitively impaired one (figure 3*e*, right), which only had a slight gradient in the proximo-distal direction. Experiments also showed more growth (larger normalized volume) in both condyles of the control group with respect to the GSK101 group (figure 2*e*, left).

Certainly, our model points to a relationship between the fluid pressure distribution and the shaping of the joint. Chondrocytes might not be sensing interstitial hydrostatic pressure directly, but rather a different biophysical factor related to it. Osmotic stresses have been repeatedly identified as the stimuli triggering a series of signalling events in relation to the TRPV4 channel, that are propagated into changes in gene expression and ECM synthesis. Yet, studies have shown that osmotic loading as well as mechanical loading elicit responses of the TRPV4 channel [24,28,29,31]. Recent publications suggest TRPV4 is a cell volume sensor and is activated regardless of the molecular mechanism underlying said volume change [69]. Furthermore, hydrostatic and osmotic pressures have similar effects on cartilage formation [70], and they both affect intracellular ion signalling in chondrocytes [71,72]. It is not within the scope of this study to determine the complex interrelations between the osmotic and hydrostatic pressures induced by mechanical loading on cartilage. Many studies have shown that hydrostatic pressure increases cartilage gene expression and extracellular matrix formation (see review in [73]). Our computational results indicate that fluid pressure can predict local tissue growth in the experimentally informed model of joint morphogenesis developed in this study.

### (d) Poroelasticity can be used to explore how dynamic loading dictates bone rudiment morphology

Owing to the nature of the poroelastic tissue, compressive dynamic loading generates the non-homogeneous fluid pressure pattern within the humerus that dictates tissue growth in our computational model (figure 3*b*). By contrast, static loading generates an initial pressure distribution that quickly dissipates as fluid seeps out of the bone rudiment (electronic supplementary material, figure S12C). Such behaviour is in agreement with experimental studies showing that cartilage growth is promoted by repetitive compressive loading while static loading inhibits cartilage growth [38,–42]. Unlike our previous models of joint morphogenesis [35,36], we are now able to inherently capture the effect because of the type of loading imposed owing to the biphasic approach that incorporates the fluid flow component into the modelling. An earlier computational study [8] used poroelasticity to relate local patterns of biophysical stimuli to the emergence of joint shape in a model of a chick knee, but could not predict growth morphologies. Through the solid component growth, our model goes a step further and can more confidently relate local tissue growth to final bone rudiment morphology based on cyclic loading-induced mechanical stimuli.

We explored alternatives to the compressive pore pressure as mechanical stimuli for our growth model (electronic supplementary material, S10), including measures of solid compression and pore fluid velocity. The positive divergence of the seepage velocity (electronic supplementary material, figure S14A, top row) stood out because, unlike the other measures, its distribution within the humerus is quite different from the fluid pressure pattern. Hence, we implemented this measure of the rate of solid compression as an alternative mechanical growth stimulus in our formulation. The resulting local tissue growth owing to the mechanical contribution was distributed more evenly towards the distal part of the humerus (electronic supplementary material, figure S15A), instead of being localized below the radius contact loading (figure 3*c*). In addition, less growth was observed in the proximal part of the humerus for the alternative model. Interestingly, this produced an apparent rotation of the humerus grown surface (electronic supplementary material, figure S15B) rather than the slight bending and outward growth observed broadly around the ventral condyle region for the pressure-based mechanical growth (figure 3*d*, left). Further study would be required to ensure artefacts owing to inadequate loading or boundary conditions are not at play here before discarding the rate of tissue compression as a potential biophysical stimuli within the joint morphogenesis process.

These exploratory simulations demonstrate the potential of the proposed model as a tool to unravel the mechanisms at play in the shaping of the joint. Through the computational study of how different measures of pressure, compression and fluid flow evolve in response to loading set-ups representative of *in vivo* conditions, we could identify potential biophysical stimuli for further study in experiments.

## 5. Conclusion

Normally regenerating axolotl forelimbs were compared to those of animals that were administered a TRPV4 agonist during joint morphogenesis, demonstrating that the TRPV4 channel has a role in the shaping of the joint. To link TRPV4 desensitization to impaired mechanosensitivity in chondrocytes, we developed a poroelastic model of joint morphogenesis. Computational results indicated fluid pore pressure is a reasonable predictor of local tissue growth and may influence local joint shape. The computational model presented provides a tool to explore alternative mechanical stimuli that may also be critical in joint morphogenesis, such as static loading or constrained conditions.

Integrating experiments and computational modelling provides interesting insights that experiments alone cannot deliver. The combined approach presented in this work allowed us to validate the mechanical regulatory hypotheses with an *in silico* model. Such methodology will become indispensable as we advance in the study of mechanobiological processes like those involved in joint formation.

## Data Availability

The original microscopy data are available on Northeastern University’s Digital Repository System at http://hdl.handle.net/2047/D20427306. The scripts and some example files used in the experimental data analysis pipeline are available on the Zenodo repository, accessible at https://doi.org/10.5281/zenodo.5591983. The computational code and simulation input files can be accessed at https://github.com/ecomellas/CompLimb-biomech.git. The data are provided in electronic supplementary material [74].
